# Chlorurets (Chlorides) of Soda and of Lime

**Published:** 1827-04

**Authors:** 


I. *On the Use of the Chlorate of Soda, and the Chlorate of Lime.* By A. G. Labarraque. Translated by James Scott, Surgeon. Octavo, pp. 36. Highly, Fleet Street, 1826.

II. *An Essay on the Use of Chlorurets of Oxide of Sodium and of Lime, as powerful Disinfecting Agents, and of the Chloruret of Oxide of Sodium more especially, as a Remedy of considerable Efficacy, in the Treatment of Hospital Gangrene; Phagedenic, Syphilitic, and ill conditioned Ulcers; Mortification; and various other Diseases.* Dedicated by Permission to the Right Honourable Robert Peel. By Thomas Alcock, Member of the Royal College of Surgeons in London; Member of the Medical and Chirurgical Society, &c. &c. Octavo, pp. 152, with a Plate. Burgess and Hill, 1827.

It is now some thirty or forty years since the fumes of nitre and salt, as disinfecting agents, were sold to the good people of England by Dr. Carmichael Smith, at a very handsome sum of money—the price being fixed by the same "collective wisdom," that gave a magnificent reward of 5000 pounds to Mother Steevens for some calcined egg-shells. At the time we mention, it was usual to see the wards of hospitals, the decks of ships, the floors of prisons—nay, the private chambers of the sick, ornamented with rows of pipkins exhaling the magic fumes that were supposed to annihilate those invisible beings called contagious or infectious *miasms*. True it is, that these acid fumes did render the air of the wards of hospitals and other domiciles of the sick, much less repugnant to the olfactories; and we had reason to believe that the confidence inspired by the new disinfectant among nurses and attendants, did, in some degree, check the spread of contagious diseases. But it was found, in the end, that, to correct a fetid smell, and to destroy a febrific or contagious miasm, were two different processes, not equally performed by the fumes of nitro-muriatic acid. The new disinfectant was soon exchanged for pure atmospheric air; but not before Dr. Smith had realized the motto on his crest—"FUMUM VENDERE."

The world is now grown older, if not wiser than it was, and specifics are put forth with more modest pretensions, and more in their true character than formerly. But the history of mankind, and especially of medicine, warns us to distrust the first accounts of extraordinary powers or virtues in medicinal substances—not because the experimenters wish to deceive; but because they are, themselves, liable to be deceived. "Experientia fallax."

It appears that M. Labarraque was led to the discovery of the powers of the substances under consideration, while endeavouring to destroy the infectious odour, and prevent putrefaction in the materials used for the manufacture of catgut and other strings made from the intestines of animals.

The CHLORURET of LIME is only a new name for the old oxymuriate of lime; and the dry preparation is a *sub-chloruret of hydrated lime*, and is well known in the arts of this country by the term BLEACHING POWDER. Of the preparation of this we shall say nothing, as few will take the trouble of preparing it, when it can be procured so cheap from the great manufactures. The formula for the chloruret of soda—or, as Mr. Alcock terms it, the "chloruret of oxide of sodium," we shall give from Mr. Scott's pamphlet, as the shortest.

"CHLORATE OF SODA.

Pure Carbonate of Soda, 2½* Kilogrammes.
Distilled Water, 10 Kilogrammes.

"Mix in a Bottle that will be about one quarter empty—then into a Glass Balloon Bottle (of about Two Quarts) with a long neck and a large mouth, put the following mixture.

Hydro-Chlorate of Soda, 576† Grammes,
Peroxide of Manganese, powdered, 448 Grammes.

"In the mouth of the Balloon Bottle, lute a large curved tube, and one also in the shape of an S; the first tube is inserted into a bottle containing a small quantity of water, a similar tube passing from this into the Bottle containing the saline solution. The lutes being dry, a mixture of 576 Grammes Sulphuric Acid, and 448 Grammes water, is to be poured into the Balloon through the curved tube S, heat is next applied and gradually regulated until the disengagement of Chlorine gas ceases.

"TEST.

Powdered Bengal Indigo, one part.
Concentrated Sulphuric Acid, six parts.

"Mix and add Nine Hundred and Ninety-three parts of distilled water—one part of the Chlorate of Soda, mixed with Eighteen parts of this liquid, should deprive it of colour.

"The saline solution should be of the specific gravity of 12°‡ by Baumè's hydrometer—if the liquid be too concentrated, add the requisite quantity of water; if, on the contrary, the solution is too weak,

* A Kilogramme is 2lb. 3oz. 5dr. Avoirdupois.

† A Gramme is about 15½ Grains.

‡ 1,089 of common standard, temperature 55, Fah.

add as much of the carbonate of Soda as will bring it to the proper strength." 36.

It is from Mr. Alcock's work that we shall draw principally in this article, that zealous and intelligent surgeon having not only witnessed the powers of the chlorurets in Paris, but also in his own practice, public and private, in this metropolis.

It appears that the disinfection of corpses was proposed to Government by Labarraque, by means of the chlorurets, and the council of health adopted his suggestions. The expense of conservation of the dead bodies in the MORGUE (a receptacle for bodies found dead, &c. in Paris, till claimed) did not exceed, in a whole year, the sum of 150 francs. Labarraque then made experiments upon living bodies, in the hospitals of Paris, and furnished the medical officers of these establishments with gratuitous supplies of the disinfectants. In 1823, he proposed plans for rendering healthy the lazarettos, and "shewed the possibility of rendering the propagation of contagious or infectious diseases impossible." A favourable report was made at the end of a year—and a prize of 3000 francs was decreed to the author by the Royal Institute.

"The disinfection of dead bodies has been proved many times, after inhumations of several days, of several weeks, and of several months, and always with the same success. The corpse of Louis XVIII. was presented to the public *without any odour*, the disinfecting chloruret of M. Labarraque having been used." P. x.

Medals and honours of various kinds now began to shower down upon M. Labarraque's head, and the cross of the Legion of Honour was soon suspended on his breast. Should the Government of this country take up the subject, in the same manner as that of France, Mr. Alcock has laudably offered to conduct a train of experiments demonstrative of the disinfectant powers of the chlorurets. We hardly expect that ministers will turn aside from their politics for the prosecution of the sciences. They certainly "manage these things better in France." *There*, the hospitals and scientific institutions are more immediately under the control and management of Government, and rewards and honours are consequently more frequently dispensed to men of merit. *Here*, there is no such system. Every thing is left to private enterprise, and such returns as the public itself may think fit to award.

Mr. Alcock has endeavoured, in this little work, to collect the scattered information relating to the subject, and to add such further observations as his personal experience has afforded. For this attempt we think he deserves the approbation of every well-wisher of medical science.

M. Labarraque observes, that it is a fortunate circumstance to be able to arrest animal decomposition, and thus, as it were, to annihilate several causes of death—but it is a still more fortunate discovery, the power of arresting decomposition in the living body. "I have had the happiness to observe this *prodigy* performed by the application of the chloruret of oxide of sodium (chloruret of soda) to wounds." Sincerely do we hope that this *prodigy* will be often witnessed in this country. But we have lived long enough in the world, to have little faith in prodigies—and especially in medical prodigies. We think the powers of the disinfectants, as to the arrest of decomposition in the dead body, however, as very fairly established by what took place at the Morgue. To this mournful receptacle all the bodies found in the Seine, and in Paris and its environs, are brought for inspection. "Even when bodies have been far advanced in putrefaction, the affusion of a solution of chloruret of lime in water has immediately removed the putrid odour." An apothecary at Lyons writes that, during the month of July, 1825, the thermometer was above 90°, and the corpses, a few hours after death, spread an odour so infectious, that during the mass which was celebrated for the repose of their souls, the priests and the friends forgot—the former the resignation of their ministry, the latter their affliction—to complain and to close their nostrils. There was fear of an epidemic, and some of Labarraque's solution was ordered to be thrown upon the sheet when the coffin was opened, to ascertain the presence of the corpse. The result was most satisfactory.

"The mode of employing the chloruret of lime to prevent or retard putrefaction is very simple, and may be effected at a trifling expense.

"To one part of chloruret of lime add forty parts of water: mix them thoroughly, and pour off the clear liquid.

"With this liquid the entire surface of the body should be freely sprinkled by means of a watering-pot, or in any other convenient manner. This sprinkling should be repeated twice or oftener, daily, according to the temperature, degree of putrefaction, &c.

"Should putrefaction be far advanced, or the period the corpse is intended to be kept be considerable, it is better to surround the body with a sheet moistened in the solution, and to renew the moistening of it frequently. By these means all putrefactive odour in the apartment may be perfectly obviated." 4.

Another important occasion, on which the chlorurets may be employed, is when the body of any distinguished personage may be required to lie in state. Mr. A. informs us that the people of Greece were deprived of the pleasure of beholding the corpse of Lord Byron, in consequence of putrefaction; while that of Louis XVIII. was approached by his subjects with safety, and without any disagreeable odour being exhaled. Another purpose is that of enabling medical men to examine bodies that have been interred, when suspicions arise as to the cause of their death. We published the account of Orfila's examination of a body that had been buried thirty-two days, and in the hottest season of the year. When the grave was opened, the odour was insupportable; but aspersions with solution of chloruret of lime, "produced marvellous effects," for the infected odour was quickly annihilated. In this case the presence of arsenic was detected in the stomach. *See Vol. I. of this Series, p.* 289. We shall quote from Mr. Alcock's work the following directions of Labarraque respecting the management of this disinfectant on such judicial occasions as the above.

" 'Before approaching a corpse in putrefaction, a tub should be procured in which may be put a load of water (24 litres, about 49 pints;) pour into this a flagon (half a kilogramme—1lb. 1oz. 10½dr. avoirdupois) of the chloruret of lime, and stir the mixture.

" 'Dip a sheet in the water contained in the tub, and unfold it so as to be able to withdraw it with facility, and particularly so as to be enabled to extend it very quickly over the corpse.

" 'To effect this, let two persons open the sheet and place it in the liquid, holding the ends upon the edges of the tub: let this be carried to the side of the body in putrefaction, and at the same instant let the wet sheet be drawn out of the tub and laid over the body.

" 'Soon afterwards the putrid odour ceases.

" 'If blood or any other fluid proceeding from the dead body have flowed upon the ground, pour upon this liquid one or two glassfuls of the chlorureted water; stir with a broom,—and the putrid odour will disappear.

" 'This operation, however, ought not to be thus performed whenever the liquids spilled upon the ground may become the subject of a chemical analysis: in this case the greatest quantity possible should be carefully collected; and it is when this has been effected that the disinfection of the ground should be performed in the manner above-mentioned.

" 'If the infection have spread in the neighbouring places, in the corridors, stairs, &c. the infected places are to be sprinkled with one or two glasses of liquid chloruret of lime, and the fetid odour will cease.

" 'Care must be taken to moisten frequently with the liquid contained in the tub, the sheet which covers the corpse: the reproduction of the putrid odour will be thus prevented.

" 'As soon as the body has been removed, the sheet which has served for the disinfection should be washed in large quantities of water, dried and folded.' "

The disinfectant power of the chlorurets promises to be of great advantage in the prosecution of practical anatomy, to the pursuit of which so many obstacles now present themselves. We do not say that dissection wounds are dangerous in proportion to the advance of putrefaction; but there can be no doubt that the exhalations of a putrid body must be prejudicial to the student's health, and therefore it would be very desirable that such exhalations should be corrected. But, as Mr. Alcock has justly observed, there is no royal road to such an extinction of putrid effluvia, without great attention to cleanliness and ventilation. Mr. Alcock throws out some excellent hints as to the important subject of hygiene in the dissecting-room, which we recommend to the attention of those who have the charge of such establishments.

In private dissections, it is often of importance to be able to preserve parts as long as possible, where time is fully occupied by professional pursuits.

"The mode," says Mr. Alcock, "by which I have preserved parts fit for dissection during weeks, and even months, sometimes in summer, has been to macerate them in warm water, taking care not to overheat the subject, and to inject into the arteries a solution of nitrate of potash, either saturated or very nearly so, at the temperature of from 120 to 130 degrees; to let this be retained a few minutes, and then to turn the stopcock so as to allow any part that might remain in the larger vessels to flow out; then, the temperature being kept sufficiently high to prevent the chilling of the injection, to throw in gradually a small quantity of fine injection, previously heated by immersing the vessel containing it in hot water; and lastly, to throw in rather briskly the wax injection of a proper temperature, so as to fill the larger vessels and to propel the fine injection into the capillaries. When cold, the dissection may be commenced. By these means the beautiful and florid appearance of the muscles is preserved or heightened: but the salt is apt to effloresce on surfaces which are long exposed: and if the cuticle be removed the skin becomes horny, the adipose substance oily, and, in short, in such a condition as to render the tracing of the cutaneous nerves impracticable. These inconveniences may be obviated by conjoining with the above the use of either the chloruret of lime or of the oxide of sodium—for this purpose aspersions will seldom be required. If, when the dissection is not continued, the integuments, &c. be replaced, and the parts be surrounded with cloths moistened in a solution of the chloruret, the preservation may be effected for an indefinite length of time, sufficient at least to afford opportunity for a careful and complicated dissection, even when only a very few hours of each day can be devoted to the subject."

The importance of a disinfectant, such as the chlorurets, will be easily appreciated where *pathological* investigation is to be prosecuted.

The disinfection of hospital wards, sick-rooms, &c. is next considered. And here we must be allowed to express our scepticism respecting the disinfectant powers of the chlorurets, till much more precise and specific evidence is put forth than what we find in the statements of Labarraque, supported even by the talents and discernment of Mr. Alcock. The proofs adduced in favour of the nitro-muriatic acid fumes, previously to the parliamentary reward, were infinitely stronger than those here brought forward; and yet few, if any, of those who have been in scenes of infection and contagion, now place confidence in the disinfectant properties of the said gases. We were, therefore, a little surprised that Mr. A. should have written the following paragraph.

"The efficacy of acid fumigations, and still more decidedly those by chlorine, has been *sufficiently* established; the former by the sanction of a parliamentary reward." 54.

If this parliamentary reward be a sufficient testimony, Mrs. Steevens' eggshells cannot be questioned as lithontriptics. But we apprehend that it is the experience of mankind, and not parliamentary remunerations, that must be looked to for proofs of this kind. The proofs adduced by Labarraqne are too vague to be relied on—witness the very first which he sets forth.

"M. Labarraque relates experiments made during *two nights* at the Bicêtre in eight wards, inhabited, and very infected. These wards, to the great satisfaction of the patients, and of the physician who attended them, have been purified, by means of sprinklings made with one bottle of the concentrated chloruret, diluted with thirty parts of water."

But, once for all, we object to the coupling of the words *purification* and *disinfection*. Bad smells may be corrected, and contagion may still remain. We have always upheld the doctrine, that *ventilation* is the best and safest disinfectant. To *remove* the infected air, is surely more effectual than to correct or cover a fetid exhalation floating in it, since we cannot tell what the contagious miasm is, or whether it really have any fetid odour or not. We shall take Mr. Alcock's own words in another place.

"From the preceding observations those who have not devoted themselves to the study of medicine might be led to suppose that the processes of disinfection before pointed out should be all that might be required to render salubrious the foul wards of hospitals and other places occupied by the sick; this would be an unfortunate conclusion, for the fact is far otherwise: although the pestilential effluvia diffused in such an atmosphere be capable of immediate correction, yet unless the cause be removed, the regeneration of similar effluvia is not prevented. Hence, therefore, the most rigid attention to cleanliness and appropriate ventilation is essential to the welfare of the sick; for although the chlorurets may destroy the putrid miasmata, they cannot furnish that supply of pure air, without which health cannot be sustained, nor disease be successfully treated. Many persons are in the habit of using fumigations of vinegar, of aromatic pastiles, and of diffusing perfumes to cover any bad smell which may pervade the sick chamber; these practices are however founded in error; *for if due attention be observed with respect to cleanliness and ventilation, not only will the accumulation of disagreeable effluvia be prevented; but the apartments of the sick will be kept as sweet as any room in a well regulated dwelling house; there is no perfume equal to a perfectly pure atmosphere, and the best test of that purity is the total absence of any odour whatever.*" 65.

In these sentiments we perfectly agree. *With* cleanliness and ventilation there will be little or no danger—*without* these, we should have no confidence in the chlorurets, the fumes, or the perfumes. Where the "pure atmosphere" cannot be put in requisition, we have no objection to the chlorurets, which may lessen or destroy fetor, inspire some mental confidence in the agents employed—and, perhaps, lessen the virulence of contagious miasms, though of this specific power we are far from having sufficient proofs.

In respect to the purification of putrid water—the disinfection of work-shops—the purification of stables—the disinfection of privies, drains, reservoirs, &c. we can say nothing from observation; but are bound to believe, from the statements of Labarraque and others, that the chlorurets are useful correctors.

There are some circumstances occasionally let out in these documents and statements which have tended somewhat to lessen the high estimation in which we were disposed to contemplate the character of Labarraque as a "distinguished philanthropist"—the term applied to him by Mr. Alcock. It appears that the composition of the disinfectant in question was kept a secret for a considerable time by Labarraque, and names given to it to disguise or veil its real nature. And why? Because Labarraque feared "the *islanders* would be able to rival and *undersell* his countrymen in this manufacture!"

"If this manufacture," says he, "were known in Great Britain, no doubt these islanders would soon endeavour to deprive us of a product which they would export in great quantities to Spain and Portugal, &c."

Self-love and social are said to be the same; and we suspect that the philanthropy of Labarraque, like charity, began at home. Mr. Battley may now adduce a *philanthropic* reason for concealing the composition of the liquor opii sedativus, lest the *Continentalists* should rival and *undersell* his countrymen in this manufacture. It is wonderful how few things, in this world, go by their proper names.

The following passage will make the inhabitants of London and Edinburgh too proud.

"In Paris, the general want of sewers renders the emptying of reservoirs of excrements a frequent and abominable nuisance, which is sometimes inflicted upon the inhabitants of the most respectable hotels, without the slightest attention to the disinfecting process which M. Labarraque has pointed out,—so little is a man a prophet in his own country.

"An English gentleman at one of these hotels observed, on returning home at night, some old dirty carpets hung up at the bottom of the staircase, and some ill-looking fellows lounging about. He could not proceed to his apartment without passing this barrier, and inquired what it meant. The answer was, '*pour empecher le mauvais odeur:*' But as there was then no bad smell, the work not having commenced, he did not anticipate what was to follow. He went to bed, but was awoke by an intolerable and suffocating stench. He got up to open his window to admit fresh air: but far from finding relief, the tubs which contained the filth occupied the street just under the window of his apartment, which was the *entresol* (or room between the parlour and the first floor), and the evil was so much increased that sleep became impossible, and he really thought he should be suffocated before morning: the thorough impregnation of clothes, furniture, and every thing in the room was the least part of the infliction; for he suffered a severe attack of bronchial inflammation, which rendered him unable to pursue his avocations for several days." 38.

It is affirmed that a train of dry chloruret of lime placed under the doors, upon such occasions, will destroy or neutralize these mephitic exhalations. As there is a very large manufactory of this disinfectant in Glasgow, we hope the good folks of "AULD REEKIE" will expend a couple of baubees weekly in chloruret of lime, for the *nether* regions of their mansions. Indeed a sprinkling of Labarraque's wash would do the streets of the modern Athens no harm, now and then, on a hot summer's morning. We strongly recommend the commissioners of sewers and the directors of gas-works, in this metropolis, to employ the chloruret whenever they open drains or mend the gas-pipes in the streets. The hydrogen effluvium which now salutes the olfactories at every turn in London, is little less abominable than the "*mauvais odeur*" which gave Mr. Alcock the bronchial inflammation in Paris.

An interesting account is given, from Labarraque, of the perilous enterprize which was undertaken, under the aid of the disinfectants, to cleanse an abominable drain, called the EGOUT AMELOT, in Paris. Several workmen had been asphyxied in former attempts—and the sewer had been "infected and impracticable for more than forty years." It was completely cleansed under the guidance of Labarraque himself, without any sinister accident.

After alluding to the observations of M. Lisfranc and Labarraque, respecting the power of the chlorurets over the poison of *small-pox and measles*, Mr. Alcock indulges in the following philanthropic anticipations.

"The beneficial results already obtained in arresting the ravages of infectious diseases, afford a well grounded presumption that the chlorurets may also be effectual in preventing the ravages of the plague, the yellow fever, and other pestilential disorders; and perhaps the period is not far distant when these dreadful scourges will no longer be dreaded, and that the first benefit from the use of such powerful disinfectants, namely, that of allowing patients labouring under these pestilential conditions to be approached with as much security and confidence as when afflicted with the ordinary diseases of our own climate, which under certain unfavourable circumstances are known to be communicable from one individual to another, may subsequently lead to improved modes of treatment, which shall in a great measure divest even the plague itself of its terrors." 59.

We shall say nothing to check such fond hopes; but proceed at once to the application of the chlorurets as remedial means in certain *diseases* to which the living body is subject. We understand that Mr. Alcock has put these means to the test of experience, and, therefore, his observations are the more valuable.

Mr. Alcock sets out with the following assertion—namely, that the chlorurets have not only the power of "destroying the most putrid effluvia arising from animal substances," but also, "the property, when applied to the substances giving off these effluvia, of arresting or destroying the progress of putrefaction." This *progress* of putrefaction, however, refers to the *dead* parts; but a little farther on, Mr. A. informs us that the application of the chlorurets generally "promotes the more speedy separation of the dead parts from the living, than can be obtained by ordinary means."

"It very often is capable of changing the nature of malignant, corroding, and destructive sores, into the condition of simple ulcers: in many ulcers not malignant, it is capable of greatly hastening the cure. In short, though not an infallible remedy, it is capable, under the guidance of medical and surgical skill, sound judgment and experience, of alleviating, and often of totally removing some of the most distressing and loathsome diseases to which the human body is liable; diseases which too often, uncontrolled by remedies previously in use, have hurried numerous victims to untimely graves." 68.

These sanguine hopes, are, however, considerably damped in the same page, by several observations tending to shew that it will be necessary for the surgeon to bring to the aid of his patient all the resources of his art, and abstract from him every thing that may prove hurtful. Under such regulations, directed by such a man as Mr. Alcock, we have no doubt that the chlorurets would be as successful as could be well expected; but where every other auxiliary was put in requisition, it would not be very easy to say exactly how much was due to the chlorurets.

The subject of hospital gangrene is prefaced by an extract of some pages from John Bell's principles of surgery, exhibiting a "vivid representation of verminous ulcer and hospital gangrene." Extracts are then given from the reports of Labarraque, and also the communications of several continental surgeons in foreign Journals, shewing the good effects of the chloruret of soda in gangrenous and other ill-conditioned ulcers. M. Cloquet, in the Hospital St. Louis, has caused the mortified limb, in several of these extremely severe diseases, to be bathed in the chloruret, diluted with ten or fifteen parts of water, and has given internally from twenty-five to thirty drops of the chloruret in a pint of ptisan. Testimonies are also adduced from Margolin, Sègalas, and many other surgeons, in favour of the remedy in question. At page 84, we find a case of sloughing of the scrotum from extravasation of urine, after bursting of the urethra, detailed from the practice of the Westminster Hospital. The man died. "The chloruret was not used." We are entirely at a loss even to guess at the reason which induced Mr. A. to introduce this case, unless it was to reprehend the neglect of using the chloruret. But men should hardly be censured for exercising their own judgment as to the propriety or impropriety of using a new remedy in a dangerous case, until the utility of the remedy be fully ascertained. This is certainly not yet the case in England.

Mr. A. informs us that Mr. Ollier, surgeon to the Western Dispensary, used the chloruret of soda with great advantage, in a case of gangrene of the cheek, in a boy. The dead parts had separated, leaving a great part of the lower jaw perfectly denuded. "There was a copious and offensive discharge, which must have tainted the air respired by the patient. A solution of the chloruret, in the proportion of one part to six of water, was applied to the ulcerated surfaces, and the dressings moistened with the same solution, from time to time: the putrid odour immediately disappeared." Mr. A. has himself used the chloruret of soda in cases of phagedenic ulcerations of the genitals, "with decided advantage."

In chronic and ill-conditioned ulcers of the legs, Mr. A. avers that the chlorurets are very beneficial, especially when the constitutional disposition on which these ulcers so frequently depend, is carefully attended to.

"The author has used both the chloruret of lime and that of soda as well in the treatment of common ulcers as in those of long standing, and has found the healing process to advance with greater certainty than under the use of the usual applications. When there is much inflammation the use of the chlorurets produces too much irritation to be proper. Sometimes the solution of the chloruret has been combined with the use of cataplasms; but more generally with common dressings, varying the support afforded to the limb according to circumstances." 96.

And again:—

"The strength of the application should be regulated so as to avoid giving any considerable pain. From three to six proportions of distilled water to one of the concentrated solution, will suffice for ordinary use; but sometimes its immediate application undiluted when the surface is very foul, may be made not only without injury, but with decided benefit. I have witnessed on several occasions the change from a foul grey surface to a clean florid appearance in twenty-four hours, and the relief to the patient's feelings correspond with the alteration in the appearance; but it is not any sudden improvement, which can supersede the necessity of strict and persevering attention: I have known patients inflict upon themselves by a single awkward application of the roller a degree of injury, which has not been recovered from in the course of a month." 97.

In some diseases of the bladder and urinary organs, the urinary secretion acquires a peculiarly offensive odour. This may be prevented, it is said, by putting a few drops of the chloruret of soda in solution, into the chamber pot. In ulcerations of the uterus, it is of great consequence to correct the fetor if possible. For this purpose the chloruret of soda should be diluted with twelve or fifteen parts of water, or even more, and then used as an injection. In this way the fetid discharge from polypus uteri may be corrected.

"Dr. Elliotson, physician to St. Thomas's Hospital, has used the chloruret in a case of diseased uterus which he had previously considered to be almost hopeless: the patient is now in a fair way of recovery." 107.

Burns and scalds are said to be treated with great success in La Pitié, by means of the chlorurets.

"*Cancer* has been disinfected, and experiments are continued on this frightful malady as also on corroding tetters. (Dartres) Cases of the cure of scald-head have equally been communicated to the Royal Academy of Medicine.*

"*Herpes.* Dr. *Biett*, Physician to the Hospital Saint Louis, has made numerous applications of the chloruret of oxide of sodium to herpetic complaints.

"*M. Sanson*, Surgeon in Ordinary at the Hotel Dieu, has disinfected *ulcerations of the mouth*, with caries of the bones of the vault of the palate, and has suspended during some time, the ravages of this frightful malady.

"*Dr. Lagneau* has made use of the chloruret in injections for the *softening of the gums, with ulcerations*, exhaling a great degree of fetor. The condition of the patient has been ameliorated, and after each injection, the odour has been destroyed.

"*M. Reynard*, dentist, has wished to employ the chloruret of oxide of sodium, to arrest the caries of teeth, and to destroy the odour of the mouth; but he has observed, that this remedy disagreeably excites the salivary glands; and on that account, he thinks it cannot be employed for the toilet of the mouth.

"*Dr. Chantourelle* has long since employed the chloruret of oxide of sodium, diluted with ten parts of water, in two cases of putrid sore throat, (*angina gangrenosa,*) and all fetid odour, so dangerous to the assistants and to the physician, disappeared: these two cases have been communicated to the Society of Medicine of Paris. Very recently also, he has derived great advantage from the use of the chloruret taken into the stomach, in the dose of twenty-five drops in a glass of water, to destroy the disengagement of sulphureous gas, which, very greatly troubled a person poisoned by the hydrosulphuret of potash, already expelled by vomiting. His memoir, read at the Royal Academy of Medicine, has given rise to a learned report, which is noticed under the head Asphyxia.

"In *Ptyalism*, and *ulcers of the mouth*, the author has employed the solution of the chloruret of oxide of sodium, with decided benefit; also in *simple and syphilitic ulcers of the throat;* in the more severe affections of these parts in that form of angina, commonly called putrid sore throat, the relief has been almost immediate.

"In the affections of the throat, in *small pox*, in *measles*, and in *scarlatina*, the use of the chloruret will prove a most valuable auxiliary, although it would not be safe to trust to a local application only to the exclusion of other means, essential to the welfare of the patient." 111.

---

* Case of a cure of *tinea favosa*, communicated to the Royal Academy of Medicine, by *Dr. Roche*. This affection had resisted the different usual modes of treatment."

Here we must close our analysis of Mr. Alcock's work, which will doubtless obtain an extensive circulation, on account of the simplicity of the remedial agents, and the number and importance of the diseases for which they may be employed. Enough, we think, has been adduced to render it *certain* that the chlorurets of lime and soda possess strong powers of destroying the *fetor* of various disagreeable or noxious substances —but, how far they are capable of annihilating the *contagious* or *infectious* miasms of certain exhalations from animal or vegetable matters, this deponent saith not.

To Mr. Alcock and Mr. Scott, the English profession is under obligations, for laying before them the various documents respecting the said chlorurets; and we have no doubt that their labours will be rewarded by the approbation of their brethren.

